# Downregulation of epithelial MHC II expression in chronic rhinosinusitis with polyps

**DOI:** 10.1186/2045-7022-3-S2-O9

**Published:** 2013-07-16

**Authors:** Julia Arebro, Lotta Tengroth, Susanna Kumlien Georén, Ola Winqvist, Lars-Olaf Cardell

**Affiliations:** 1 Karolinska University Hospital, Division of ENT Diseases, CLINTEC, Stockholm, Sweden; 2 Karolinska University Hospital, Department of Medicine, Unit of Clinical Allergy R, Stockholm, Sweden

## Background

Inflammation in the nasal mucosa has been shown to correlate with the development of CRSwNP (chronic rhinosinusitis with nasal polyps). The mechanism behind this is not fully understood but changes in the activity of the immune system might contribute to the growth of polyps. The present study focuses on the potential role of MHC II (major histocompatibility complex class II). These cell surface proteins are of critical importance for the function of cells in the immune system and potentially in nasal polyp development.

## Methods

Biopsies from polyps were obtained from patients with CRSwNP (with and without local steroid treatment during at least six consecutive weeks) as well as from the mucosa (concha inferior) of healthy control subjects. The obtained specimens were homogenized and subsequently analysed with flow cytometry.

## Results

Preliminary data indicates a downregulation of MHC II expression in polyp epithelial cells from patients with CRSwNP compared to healthy patients (healthy controls: 60,2**±**7,8; polyps 31,1**±**7,4; p <0,05). This reduction seems to be independent of the steroid treatment. In contrast, the level MHC II+/CD86+ on epithelial cells was downregulated in patients receiving steroid treatment but not in those without treatment. The mechanisms involved are presently investigated in mice.

## Conclusion

Expression of MHC II and its co-receptor CD86 can be seen on epithelial cells in the mucosa of healthy controls as well as within the polyps of CRSwNP patients. This suggests a role for epithelial cells in the antigen-presenting procedure. Change of the MHC II expression, altering the inflammatory activity in response to invading microorganisms, might contribute to the development of polyps.

